# Treatment of MRSA pneumonia: Clinical and economic comparison of linezolid vs. vancomycin – a retrospective analysis of medical charts and re-imbursement data of real-life patient populations

**DOI:** 10.3205/id000028

**Published:** 2017-01-27

**Authors:** Michael H. Wilke, Karsten Becker, Sebastian Kloss, Sebastian M. Heimann, Anton Goldmann, Bertram Weber, Mathias W. Pletz, Philipp Simon, Christian Petrik

**Affiliations:** 1 inspiring.health, Munich, Germany; 2 Institute of Medical Microbiology, University Hospital Muenster, Germany; 3 Pfizer Deutschland GmbH, Berlin, Germany; 4 1st Department of Internal Medicine, Center for Integrated Oncology CIO Cologne Bonn, German Centre for Infection Research, University Hospital of Cologne, Germany; 5 Department of Anesthesiology and Intensive Care Medicine, Charité – Universitaetsmedizin Berlin, Germany; 6 Center for Infectious Diseases and Infection Control, University Hospital Jena, Germany; 7 Department of Anesthesiology and Intensive Care Medicine, University Hospital Leipzig, Germany; 8 Pfizer Pharma GmbH, Berlin, Germany

**Keywords:** linezolid, vancomycin, methicillin resistant Staphylococcus aureus, pneumonia, treatment outcome, cost-benefit analysis, Zyvox®, Pfizer

## Abstract

**Objectives:** To supplement the data collected in randomized clinical trials, the present study in patients with methicillin resistant *Staphylococcus aureus* (MRSA) pneumonia was conducted to explore the clinical effectiveness of linezolid and vancomycin in a routine clinical setting. Further, the overall costs of the patients' stay in the intensive care unit (ICU) were compared.

**Methods:** This was a retrospective analysis of medical and reimbursement data of adult patients who were treated for MRSA pneumonia with linezolid or vancomycin. Since the subjects were not randomly assigned to treatments, propensity score adjustment was applied to reduce a potential selection bias.

**Results:** In total, 226 patients were included; 95 received linezolid and 131 received vancomycin as initial therapy for MRSA pneumonia. Switches to another antibiotic were observed in 4 patients (4.2%) receiving linezolid and in 23 patients (17.6%) receiving vancomycin (logistic regression analysis; odds ratio linezolid/vancomycin: 0.183; 95% confidence interval [CI]: 0.052–0.647; p<0.01). All-cause in-hospital mortality was also lower in patients receiving linezolid (22 patients [23.2%] vs. 54 patients [41.2%]) (logistic regression analysis; odds ratio linezolid/vancomycin: 0.351; 95% CI: 0.184–0.671; p<0.01). The analysis of the total costs of stay in ICU did not reveal any major differences between the two treatment groups (cost ratio linezolid/vancomycin: 1.29; 95% CI: 0.84–1.98; p=0.24).

**Conclusions:** These findings confirm in a routine clinical setting that linezolid is a valuable therapeutic alternative to vancomycin for the treatment of MRSA pneumonia. However, prospective studies in real-life patient populations are warranted.

## Introduction

Infections with methicillin resistant *Staphylococcus aureus* (MRSA) pathogens represent a substantial economic burden for the healthcare system [1], [2]. For decades, vancomycin has been the gold standard for the treatment of MRSA infections [3], [4], but during the last years, new drugs have broadened the spectrum of therapy options. For the treatment of MRSA pneumonia, for example, linezolid has been an approved alternative to vancomycin since 2001 and its use is recommended in various current guidelines [5], [6]. First indications that linezolid – due to its superior tissue penetration [7] and pharmacokinetic/pharmacodynamic index – might be not only clinically non-inferior but also superior to vancomycin in this indication were found in a retrospective subgroup analysis of two registration trials for linezolid [8], [9]. Later, a prospective, randomized, double-blind, controlled study confirmed the results of this analysis [10]; see review by Torres for a discussion of the strengths and weaknesses of this trial [11]. Further, the results of two recent modelling studies have shown that the use of linezolid is cost-effective in patients with nosocomial pneumonia [12], [13]. In a retrospective observational study, Peyrani et al. observed a higher clinical success rate in patients receiving linezolid for the treatment of ventilator-associated pneumonia due to MRSA than in patients receiving vancomycin; resource utilization, on the other hand, was not different in the two groups of patients [14]. The necessity of such “real-life” studies has recently been pointed out by Zimmermann et al., who showed – using the example of tigecyclin – that individuals potentially eligible for participation in randomized controlled trials represent only a minority of the target population for antibiotics [15]. Critically ill patients, in particular, were found to be underrepresented.

The present exploratory study in patients with MRSA pneumonia was conducted to investigate the clinical effectiveness of linezolid and vancomycin in a routine clinical setting and to compare – from a hospital perspective – the overall costs associated with the patients’ stay in the intensive care unit (ICU).

The primary data source for this retrospective analysis was reimbursement data for hospital services. This procedure followed the approach used by Wilke and Grube, who used diagnosis-related groups (DRG) data to analyze the costs of treatment of severe infections caused by multi-resistant pathogens and showed that – among other variables – the length of stay (LOS) in ICU is a valid endpoint for the pharmaco-economic evaluation of antibiotic treatments [16]. 

## Methods

### Study design

This was a retrospective database and chart review study in patients who were treated for microbiologically confirmed MRSA pneumonia in German hospitals during the 5-year period from January 2008 to December 2012. Patients ≥18 years of age with pneumonia (with or without mechanical ventilation) and the presence of MRSA as relevant pathogen were eligible for this study. Comparisons were made between patients receiving linezolid (Zyvox^®^, Pfizer) as initial therapy for MRSA pneumonia and patients receiving vancomycin as the sole MRSA-effective drug. The variables analyzed were

1. the length of stay (LOS) in ICU after start of MRSA therapy,
2. the total costs of stay in ICU from start of MRSA therapy,
3. the number of patients who were switched to another antibiotic after the 5^th^ treatment day,
4. in-hospital mortality (survival status at discharge), and
5. time to death.

The total costs of stay in ICU were calculated using the German DRG system of the year in which the patient was treated. Adjustment for inflation was done on the basis of the German consumer price index for health care [17].

### Data sources and data analysis

Data sources were

1. data submitted to health insurance companies for reimbursement purposes,
2. the microbiology database of the hospital, and
3. the patients’ medical records.

All data were analyzed with descriptive statistics. Further, the total costs of stay in ICU were analyzed using a generalized linear model (GLM) with negative binominal distribution and log-link. This standard approach for the analysis of overdispersed data [18] was chosen when it was detected that the originally planned Poisson GLM with log-link was inappropriate for the data (overdispersion present). As a sensitivity analysis, the analysis of cost data was conducted not only on the basis of the full data set but also after exclusion of influential outliers. Such values were identified using the DFBETA method [19]. Switches to another antibiotic after the 5^th^ day were analyzed with logistic regression. In-hospital mortality (survival status at discharge) and time to death were analyzed using logistic regression and the Kaplan-Meier method, respectively. 

All statistical models applied included propensity score (PS) quintiles as a covariate to reduce the potential for selection bias. The PS was calculated as the probability of receiving linezolid based on the following patient characteristics:

- demographics,
- principal diagnosis,
- Patient Clinical Complexity Level (PCCL),
- type of MRSA infection (mono/mixed),
- MRSA infection before stay in ICU (yes/no),
- antibiotic pre-treatment (yes/no), and
- reason for hospital admission.

The PCCL is a basic variable in the German DRG System, which reflects the severity of comorbidities. It is computed of the Complication and Comorbidity Levels (CCL) of each resource consuming secondary diagnosis coded in the patient’s dataset. The PCCL is a discrete variable with values of 0 to 4. In addition to the PCCL, the sum of all CCLs was calculated (PCCL_native) to obtain a better understanding of the comorbidities of each patient.

Statistics were calculated using SAS^®^ v.9.2 (SAS Institute Inc., Cary, NC, USA). All analyses were of exploratory nature. 

## Results

### Study population

Data from 226 evaluable patients were retrieved from the records of 10 university and maximum-care hospitals in Germany. Ninety-five (95) of the 226 study participants received linezolid as initial therapy for MRSA pneumonia and 131 received vancomycin. The majority of patients were ≥50 years of age and male (Table 1 (Tab. 1)). The most common principal diagnoses were *disease of the circulatory system* and *disease of the respiratory system*; a principal diagnosis of MRSA pneumonia was relatively uncommon. Comorbidities and complications were the rule; almost all patients (94%) had a PCCL of 3 or 4, i.e. a relatively high score, which indicates the presence of complications or co-morbidities that are expected to affect the length and the costs of stay in hospital. 

### Antibiotic treatment and LOS in ICU

In most cases, treatment with vancomycin or linezolid was started immediately after microbiological diagnosis or even before diagnosis (empirical therapy) (Table 2 (Tab. 2)). The mean duration of MRSA therapy was slightly longer in the linezolid group than in the vancomycin group and switches to another antibiotic after the 5^th^ treatment day were less common in the linezolid group (Table 2 (Tab. 2)). 

Most patients were in ICU for at least one day after the start of MRSA therapy (96.3%). Generally, the LOS in ICU after the start of MRSA therapy exceeded the duration of the therapy by far. The difference between the mean LOS in ICU and mean duration of therapy was 26 days in the linezolid group and 25 days in the vancomycin group. 

### Effectiveness, safety and tolerability of antibiotic treatment

**Figure 1:**
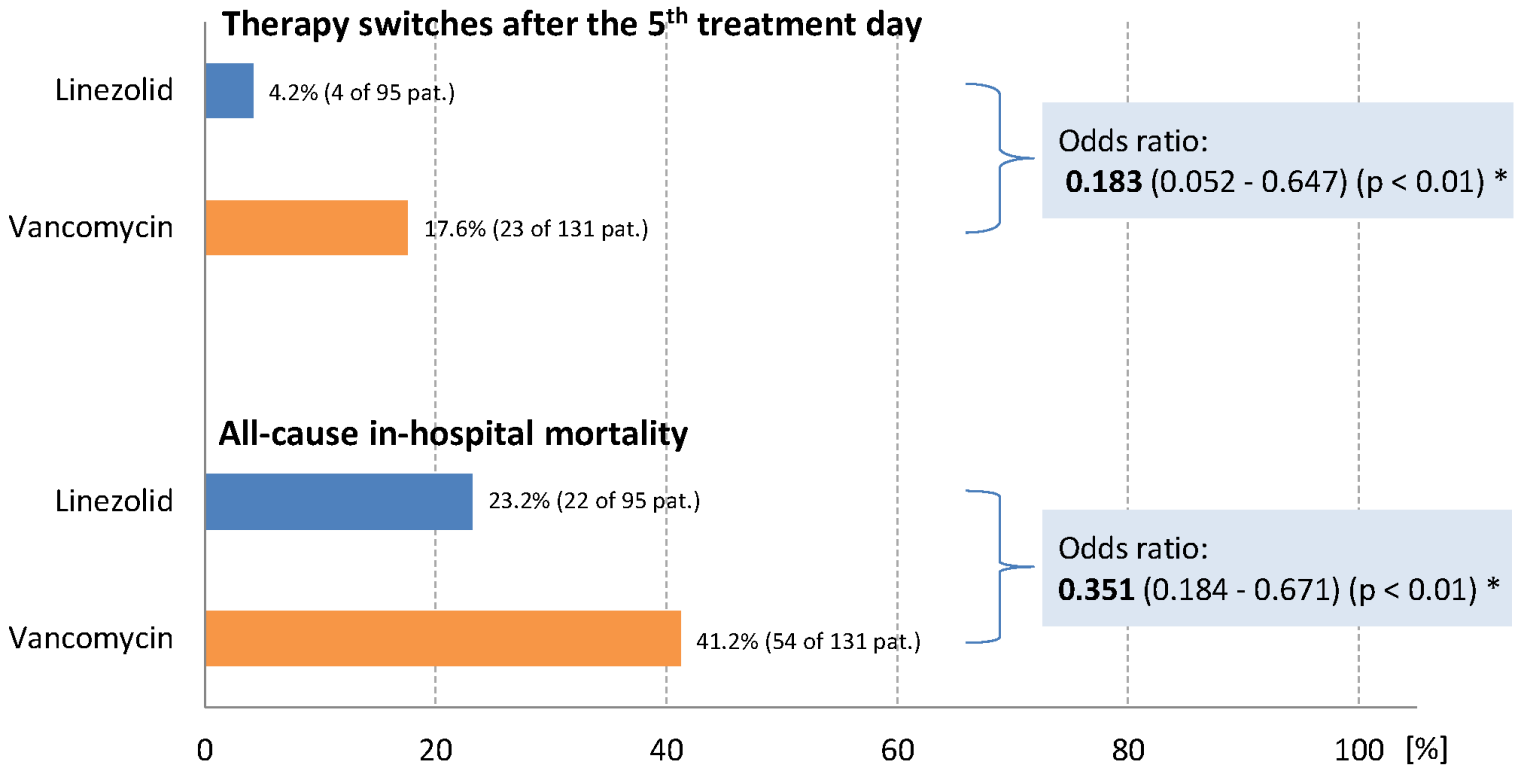
Switches to another antibiotic and in-hospital mortality rate * Logistic regression analysis; odds ratio linezolid/vancomycin (95% confidence interval). The figure shows that the risk of dying in hospital as well as the likelihood of being switched to another antibiotic after the 5^th^ treatment day (for whatever reasons) were lower in the linezolid group.

**Figure 2:**
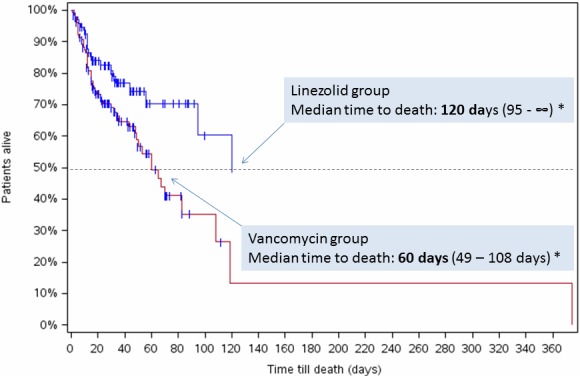
In-hospital mortality: Kaplan-Meier curves Vertical dashes indicate censored data (here: patients cured); * Kaplan-Meier estimate (95% confidence interval). The figure shows that the median time to in-hospital death was shorter in the vancomycin group.

There were obvious differences between the two treatment groups regarding in-hospital mortality and therapy switches: Both the risk of dying in hospital (for whatever reason) and the likelihood of being switched to another antibiotic after the 5^th^ treatment day (for whatever reason) were markedly lower in the linezolid group than in the vancomycin group (Table 2 (Tab. 2), Figure 1 (Fig. 1)). The estimated median time to death (in-hospital) was also considerably longer in the linezolid group (Table 2 (Tab. 2), Figure 2 (Fig. 2)). 

Indications of differences in the safety and tolerability of the two treatments were not observed: Signs of nephrotoxicity were detected equally often in both treatment groups (linezolid: 17 of 95 patients (17.9%); vancomycin: 21 of 130 patients (16.2%), 1 patient with missing data; Fisher’s exact test: p=0.723). 

### Total costs of stay in ICU 

**Figure 3:**
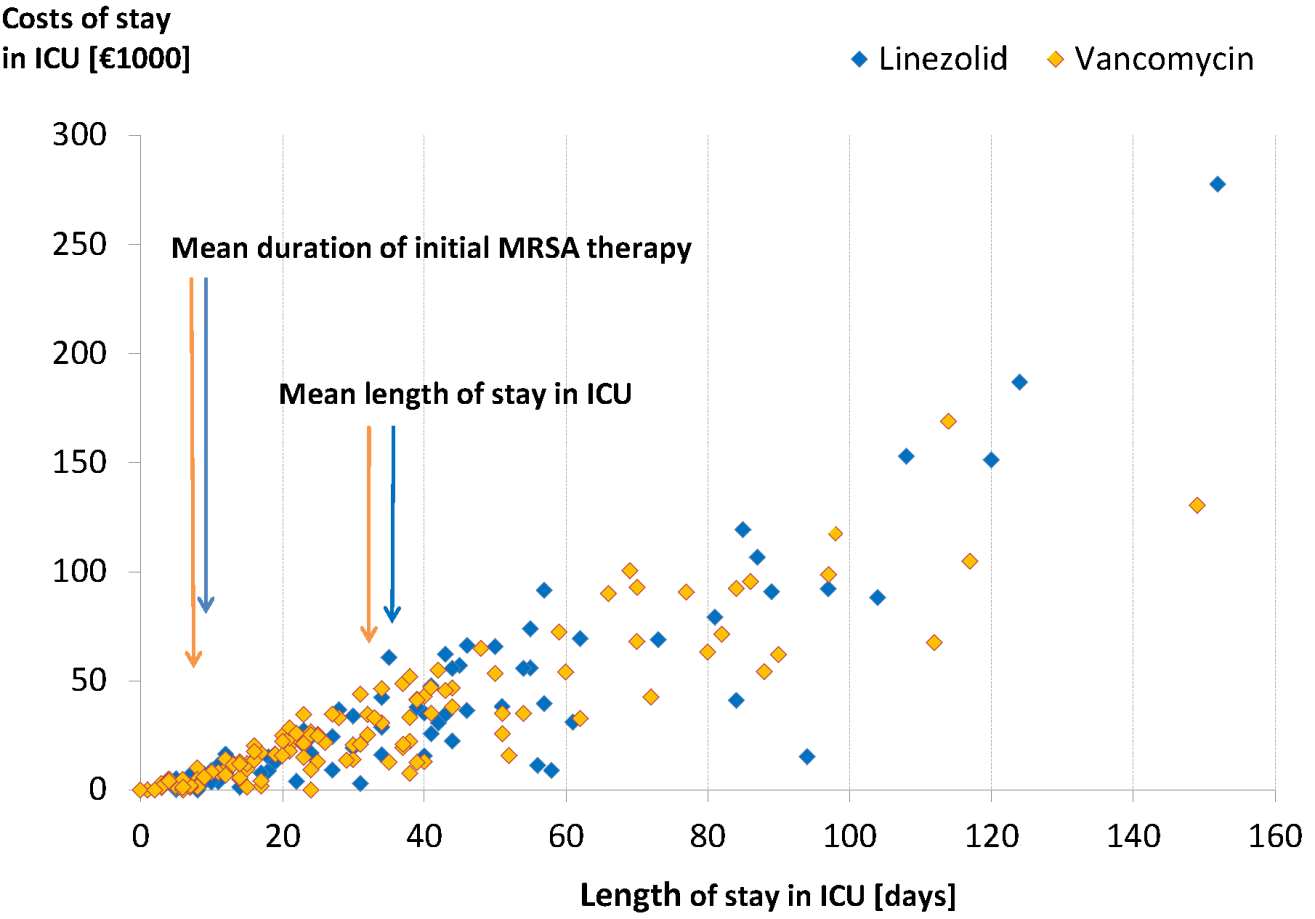
Costs of stay in ICU vs. length of stay in ICU The figure shows that the length and the costs of stay in ICU were closely correlated. Generally, the length of stay in ICU (after start of MRSA therapy) exceeded the duration of MRSA therapy by far.

The LOS in ICU was closely correlated with the total costs of stay in ICU (Spearman’s correlation coefficient r_s_=0.89, p<0.0001) (Figure 3 (Fig. 3)). The distribution of these costs was skewed to the right. It appeared to comprise two parts:

1. the “body” [20] of the distribution, which included the majority of patients, i.e. patients with low to moderate lengths and costs of stay in ICU, and
2. the tail of the distribution, which included patients with extremely long LOS and high costs.

Accordingly, mean and median costs differed markedly within the two treatment groups. The mean costs were higher in the linezolid group than in the vancomycin group (Table 2 (Tab. 2)); the median costs, in contrast, were lower in the linezolid group (€16,800 vs. €20,700). The GLM analysis of the cost data (full data set) did not reveal any significant cost differences between the two treatment groups (Table 2 (Tab. 2)). This finding is confirmed by the results of the sensitivity analysis based on outlier-adjusted data (DFBETA set). 

### ICU costs in relation to better survival odds 

By applying the different survival rates to the mean costs of stay in ICU in the two treatment groups (using observed, non-PS-adjusted data), it was possible to estimate the extra costs per life saved. For the study population as a whole, these were estimated at €314 per life saved when linezolid instead of vancomycin was used for the treatment of MRSA pneumonia.

## Discussion

This study was a retrospective analysis of medical and reimbursement data. This approach allowed a comparison of costs and effectiveness between the two treatments – linezolid and vancomycin – in a broad patient population in a routine clinical setting. Such real-life studies are essential because the individuals included in randomized controlled trials with a drug might represent only a minority of the intended target population as shown by Zimmermann et al. [15]. In the present study, the hospitals submitted the data of all their patients who met the inclusion criteria, i.e. received linezolid or vancomycin as an initial treatment for MRSA pneumonia in the 5-year study period. In consequence, the population studied covered the full spectrum of disease severity. However, it has to be taken into consideration that the sample size was relatively small. 

A frequently discussed limitation of retrospective chart review studies such as the present study is that patients are not randomly allocated to treatments and that thus the treatment effects observed might be confounded. For the present study, a look at the pre-treatment patient characteristics shows slight differences between the two treatment groups, e.g. regarding the male/female ratio or the frequency of the principal diagnoses “respiratory system disorders” (including a principal diagnosis of MRSA pneumonia) or neoplasms as well as the difference in all-cause mortality (Table 1 (Tab. 1)). However, these pre-treatment differences do not substantially limit the interpretability of the study results since PS quintiles based on baseline variables such as gender, age, principal diagnoses and comorbidities (via the PCCL) were included as covariates in all the statistical models applied in this study. With including these factors in the PS, the covariates were sufficiently controlled.

One of the key findings of this study was that both the risk of dying in hospital and the likelihood of being switched to another antibiotic after the 5th treatment day were markedly lower in the linezolid group than in the vancomycin group. In accordance with clinical standard procedures, such switches were interpreted as “therapy failures”. However, this interpretation can be made only with certain reservations since – due to the nature of the study – no information about the reasons for switching the patients to another antibiotic was available. The same applies – *mutatis mutandis* – to the mortality data analyzed. 

It goes without saying that in-hospital mortality had an effect on the LOS in ICU. The observed differences in the mean lengths and costs of stay in ICU that favor vancomycin were possibly caused by earlier deaths. However, attention should also be paid to the fact that the ICU cost distribution was markedly skewed to the right. Such skewedness *per se* is not unexpected, but it renders the arithmetic mean relatively useless for describing the central tendency [20] – at least as long as the full data set is analyzed. Weissman recommends using the median, mode, or harmonic mean in such cases. The median ICU costs observed in this study showed a clear trend (linezolid group: €16,800; vancomycin group: €20,700). The exclusion of influential outliers that were identified by the DFBETA method, a method recommended by Weichle et al. for this purpose [21] also harmonized the results and led to nearly identical average cost estimates for both groups. Worth mentioning might be that Weichle et al. observed in their study that different approaches of outlier exclusion yielded similar results with regard to the average cost estimates. This observation could be replicated on the basis of the present data [22]. 

## Conclusions

We conclude that the data analyzed confirm – in a routine clinical setting – the clinical response results of the retrospective subgroup analyses of the two registration trials for linezolid [8], [9] as well as the results of a retrospective observational study [14] and a prospective randomized, double-blind, controlled study [10]. In summary, it can be concluded that patients who received linezolid for the treatment of MRSA pneumonia had a lower risk of being switched to another antibiotic after the 5^th^ treatment day – a switch, which, with all due reservation, can be interpreted as an indication of therapy failure – and a lower risk of death compared with patients who received vancomycin. This study also confirmed results of published cost-effectiveness analyses, which showed that treatment of MRSA pneumonia is cost effective compared with vancomycin. The extra drug acquisition costs for this advantage (in 2008 to 2012: approximately €100 per treatment day) can be considered marginal in comparison to the total costs of stay in ICU. 

However, further research addressing areas of different prevalence and distribution of MRSA lineages is required in broad real-life patient populations to confirm these results.

## Notes

### Acknowledgements

The authors would like to thank Thomas Fischer from Winicker Norimed GmbH, Nuremberg, Germany, for providing biometrical support, and C. Hilka Wauschkuhn, Bonn, Germany, for providing medical writing services on behalf of Pfizer Deutschland GmbH.

### Financial support / funding

The study as a whole including preparation of the manuscript was sponsored by Pfizer Deutschland GmbH, Germany.

### Competing interests

MHW and MWP have served as consultants to Pfizer Deutschland GmbH. KB has received lecture fees, research grants, and travel support from Pfizer. SMH has received travel grants from Pfizer. SK, BW, and CP are employees of Pfizer Deutschland GmbH or Pfizer Pharma GmbH, respectively. PS and AG have no potential conflicts of interest to declare.

### Ethics statement

The study protocol was reviewed and approved by the Ethics Committee of the Medical Association of Westfalen-Lippe and the Medical Faculty of the University of Muenster, Germany.

All patient information was anonymized prior to analysis.

## Figures and Tables

**Table 1:**
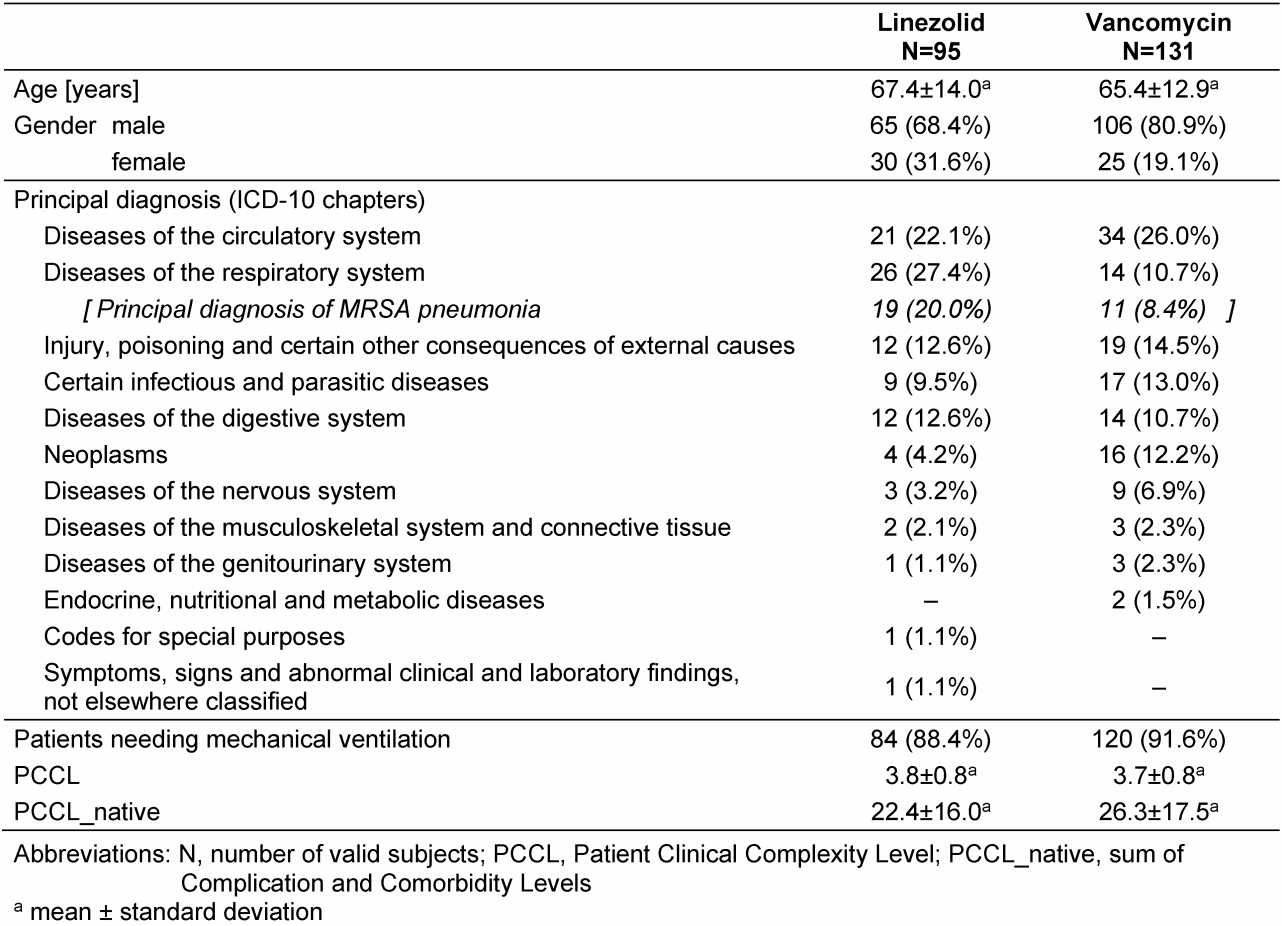
Patient characteristics

**Table 2:**
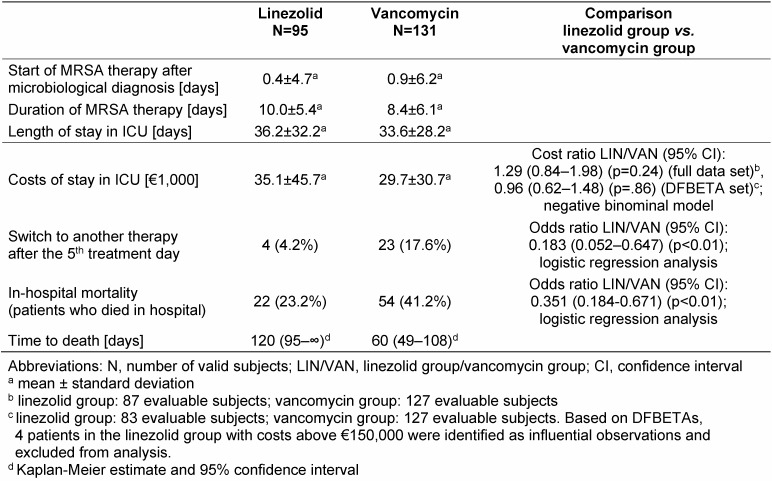
MRSA therapy, length and costs of stay in ICU, in-hospital mortality
